# Atopic dermatitis associated with systemic tacrolimus: Case report and review

**DOI:** 10.1177/2050313X251359030

**Published:** 2025-08-28

**Authors:** Sheenagh J. K. Luijk, Cathryn J. Sibbald

**Affiliations:** 1 Division of Dermatology, Department of Paediatrics, The Hospital for Sick Children, University of Toronto, ON, Canada

**Keywords:** atopic dermatitis, tacrolimus, transplant

## Abstract

A 10-month-old female was referred to dermatology clinic for evaluation of eczema, noted at 110 days after deceased-donor liver transplant while receiving systemic tacrolimus as monotherapy for posttransplant immunosuppression. She was seen and diagnosed with new onset atopic dermatitis (AD), thought to be in part related to her tacrolimus. After failing conventional treatment, her immunosuppression was switched from tacrolimus to sirolimus, and she received 1 dose of dupilumab. After 1 month, her skin had considerably improved and further doses of dupilumab were not pursued. At last follow-up, her eczema had completely cleared. Although infrequently reported in the literature, there is a growing body of evidence to suggest systemic tacrolimus may induce AD in transplant recipients.

## Introduction

The field of transplantation has rapidly evolved over the past two centuries, leading to combined surgical and treatment regimens that are life-saving. Success largely depends on balancing effective recipient immunosuppression with its potential morbidity. One of the most frequently used immunosuppressive agents is FK-506, that is, tacrolimus. Up to “100-fold more potent than cyclosporine,”^1^ it is associated with lower rates of rejection in comparison to previous preferred drug(s),^2^ and is now used in the majority of transplant cases.

Despite its beneficial effects, there are numerous reports of systemic tacrolimus being linked to the development of “de novo” atopy in transplant recipients.^3^ We present here a case of presumed tacrolimus-induced atopic dermatitis (AD) and a review of the literature.

## Case presentation

A late preterm infant, born at 36 weeks gestational age, was found to have biliary atresia and underwent a Kasai procedure on day 21 of life. She progressed to acute liver failure and required transplantation. At 4 weeks old, she received a deceased donor ABO & EBV/CMV mismatch liver transplantation. Rabbit antithymocyte globulin, cumulative dose 2 mg/kg, was used for induction immunosuppression. She was started on IV Methylprednisolone and systemic tacrolimus, targeting a trough level of 10 to 12 mcg/L.

**Figure 1.**
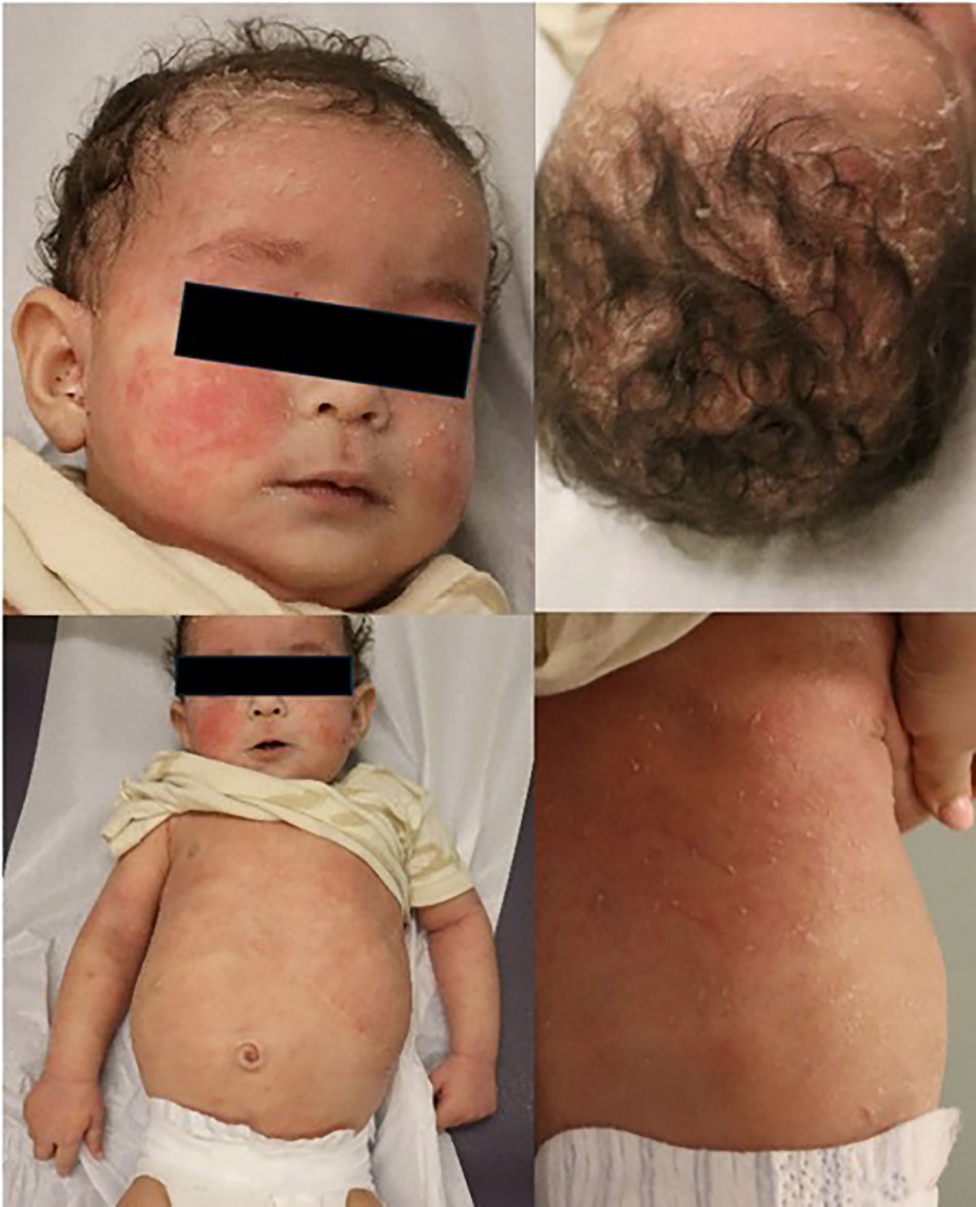
Visit #1; POD 300. Widespread involvement of ill-defined, erythematous dermatitic plaques with overlying scale and plate-like adherent scale on hair-bearing scalp. POD = post-operative day.

From as early as 4 months posttransplant, not long after stopping systemic corticosteroid, the patient developed widespread eczema and was started on topical corticosteroids from the transplant team. She was seen by the dermatology team in consultation on postoperative day (POD) 300. She was erythrodermic with severe widespread erosions and scaling (Figure 1). Management included full body betamethasone valerate 0.1% and fluocinolone oil for the scalp. Nonpharmacologic management recommendations included bland emollient, antifungal shampoo, and daily baths. At this point, a concern of possible tacrolimus-induced AD was raised.

At follow-up 1 month later, her skin had worsened with an increase in excoriations and scaling on the body and scalp. Full body mometasone was started, with continuation of her other treatments. Possible egg allergy by history prompted a referral to a pediatric allergist specialist. A strong recommendation for change from tacrolimus to sirolimus was discussed with the patient’s family and transplant team.

**Figure 2.**
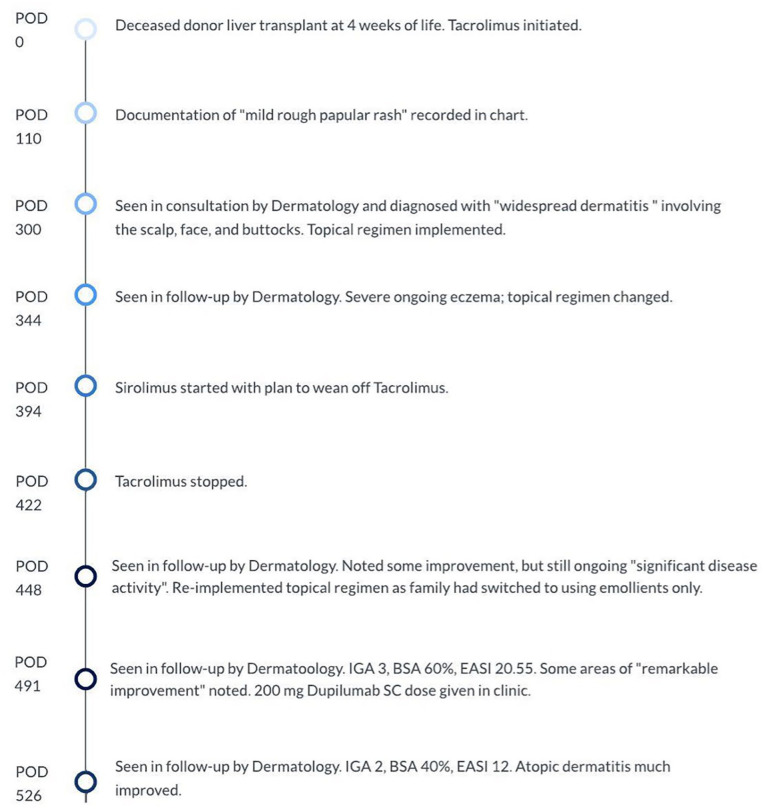
Timeline of case summary.

**Figure 3.**
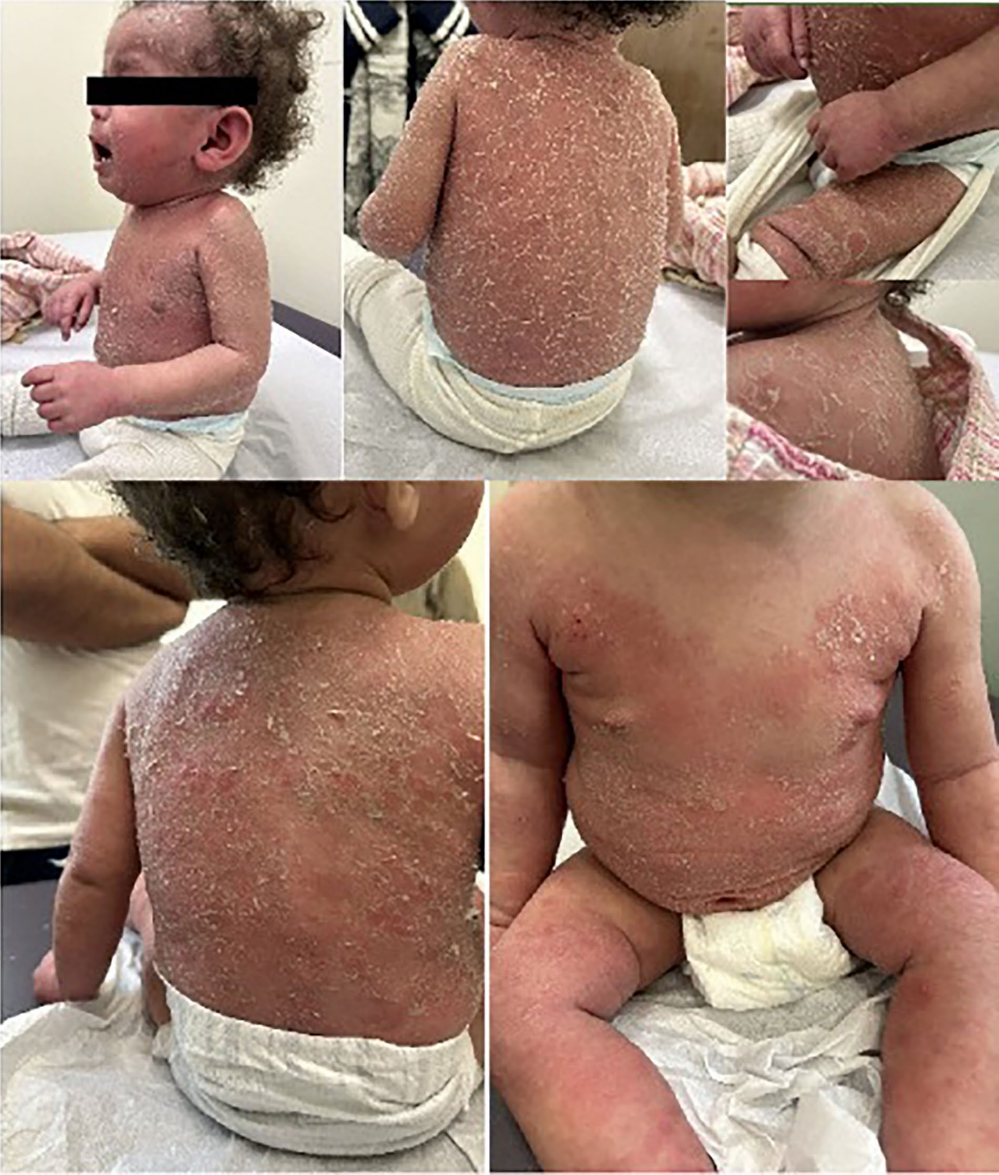
POD 448. Note the ichthyotic-like scale and diffuse erythema. POD = post-operative day.

**Figure 4.**
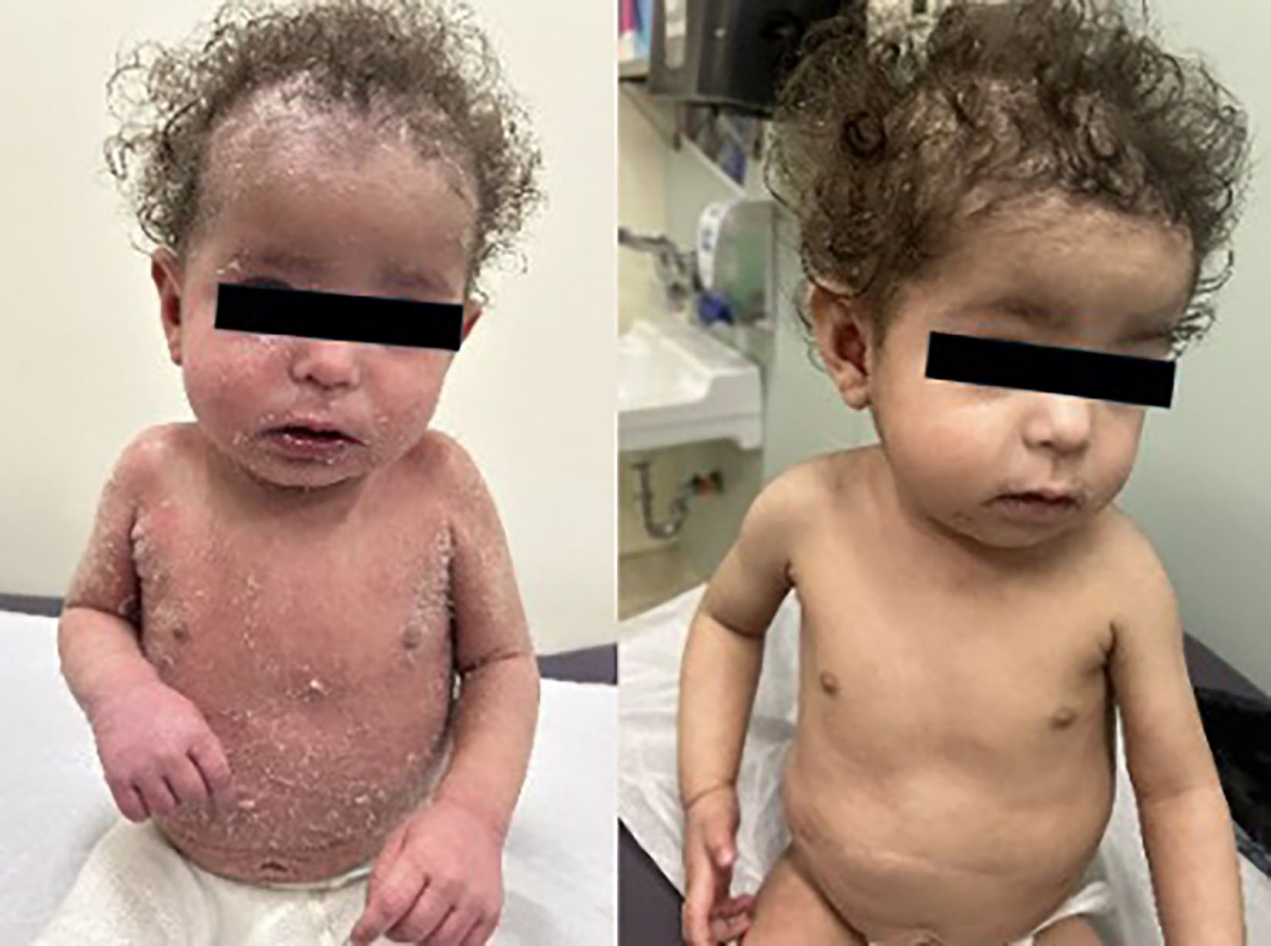
Dramatic difference in severity of skin disease between POD 448 versus POD 491; note: Tacrolimus stopped on POD 422 and Dupilumab dose given on same day as right-side photo (i.e. POD 491). POD = post-operative day.

**Figure 5.**
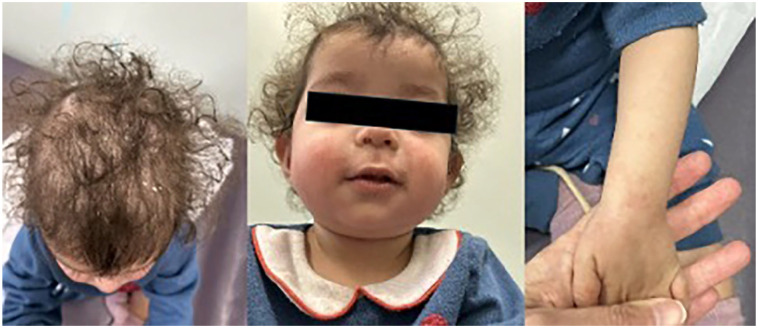
POD 526; skin is almost fully cleared. POD = post-operative day.

**Figure 6.**
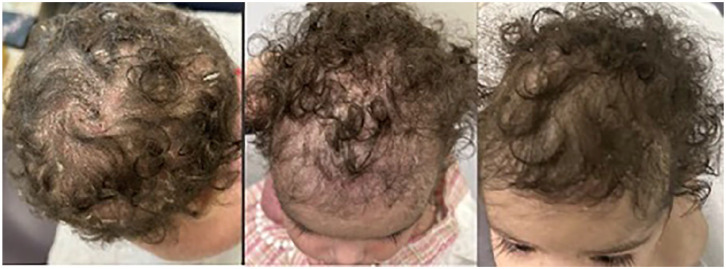
Second visit, third visit, fourth visit showing gradual clearing of erythema and thick scale on scalp.

On POD 394, sirolimus was started and on POD 422, tacrolimus was discontinued (Figure 2). The patient’s eczema gradually improved, and at reassessment on POD 491, the family estimated a 70% improvement in the patient’s skin. Despite the improvement, dermatology scoring revealed an Eczema Area and Severity Index (EASI) of 20.55(EASI score of 0.1–1 = almost clear, 1.1–7.0 = mild; 7.1–21.0 = moderate; 21.1–50.0 = severe, 50.1–72.0 = very severe), BSA involvement of 60%, and a physician global assessment of severity of 3. A single dose of Dupilumab 200 mg was given subcutaneously, an IL-4 and Il-13 receptor antagonist. Repeat dermatology follow-up occurred on POD 526 and at that time, the patient showed significant improvement. Repeat scoring revealed a significant reduction in the EASI score, down to 12, a reduction in IGA to 2, and BSA reduced to 40%. Note was made that the patient’s back, which had previously been completely involved (see Figure 3), was now entirely clear. Further doses of dupilumab were not pursued, and the patient’s skin remained clear at subsequent follow-up with minimal topical steroid use (Figures 4,5,6).

## Discussion

This case describes new onset severe AD in an infant occurring after starting systemic tacrolimus and resolving with a switch to systemic sirolimus. A literature search was performed to retrieve information on other reported associations between systemic tacrolimus and AD. The search strategy for Ovid MEDLINE (Supplemental Table S1) was modified for Ovid Embase and Clarivate Web of Science. Studies were limited to those with individual extractable data. A total of seven papers were found for inclusion (Supplemental Table S2). Across included studies, 29 patients developed new or worsening AD while on systemic tacrolimus; 2 were adults and the rest pediatric patients (age <18 years). The average time from transplant to AD diagnosis was 12.11 months (standard deviation 12.6 months). In total, 28/29 patients had nonpharmacologic and standard pharmacologic interventions implemented (bland emollients, topical steroids, etc.). In 17/29 patients, data were not recorded on immunosuppressant change. In 13 patients for whom data was recorded on immunosuppressant decision-making, 4 patients had either tacrolimus stopped or decreased. Of those, 4/4 had significant AD improvement.

Systemic tacrolimus has been implicated in causing a skewed Th1/Th2 ratio,^4^ as IL-2 inhibition shifts the body’s cytokine response toward Th2.^5^ This produces a relative suppression of Th1 lymphocytes.^6^ This predominance of Th2 cytokines, Il-4, Il-5, and Il-13, has been theorized to contribute to a rise in eosinophils and IgE^1,7^—key players in the pathophysiology of atopic disease. Immunosuppression with systemic tacrolimus has been rarely associated with de novo development or profound worsening of atopic disease (AD) in the transplant population.

In this article, we presented a case of an infant receiving systemic tacrolimus postliver transplant who developed new-onset severe AD, refractory to standard therapy. Upon switching from tacrolimus to sirolimus, her skin disease improved remarkably. More data would be helpful to identify any patient or transplant-related characteristics which may increase the risk of tacrolimus-associated dermatitis, but this case highlights a promising management strategy for patients who develop this adverse effect.

## Supplemental Material

sj-docx-1-sco-10.1177_2050313X251359030 – Supplemental material for Atopic dermatitis associated with systemic tacrolimus: Case report and review Supplemental material, sj-docx-1-sco-10.1177_2050313X251359030 for Atopic dermatitis associated with systemic tacrolimus: Case report and review by Sheenagh J. K. Luijk and Cathryn J. Sibbald in SAGE Open Medical Case Reports
