# Supplementary material for: Atopic dermatitis associated with systemic tacrolimus: Case report and review
Source: SAGE Open Med Case Rep. 2025 Aug 28;13:2050313X251359030. doi: 10.1177/2050313X251359030 (PMC12394866; doi:10.1177/2050313X251359030)
Supplement: sj-docx-1-sco-10.1177_2050313X251359030 – Supplemental material for Atopic dermatitis associated with systemic tacrolimus: Case report and review [file sj-docx-1-sco-10.1177_2050313X251359030.docx]

Table S1: Search Strategy

| **#** | **Searches** | **Results** |
| --- | --- | --- |
| 1 | Dermatitis, Atopic/ci, et, im, di or *Skin Diseases/di, dt, et, im or *Inflammation/di, dt, et, im or ((Atopic or infantile) adj3 (Dermatitis* or eczema* or neurodermatitis*)).mp. | 80353 |
| 2 | Tacrolimus/ad, ae, tu, pk, to, tu or *Immunosuppressive Agents/ae, tu | 40905 |
| 3 | (systemic* adj3 (Tacrolimus or Prograf* or FK-506 or FK506 or FR-900506 or FR900506)).mp. | 226 |
| 4 | (tacrolimus adj2 (withdraw* or discontinu*)).mp. | 297 |
| 5 | or/2-4 | 41083 |
| 6 | 1 and 5 | 1271 |
| 7 | "Drug-Related Side Effects and Adverse Reactions"/ or (chemically-induc* or drug react* or side effect* or adverse event* or adverse effect* or sequel* or toxic*).mp. | 3853043 |
| 8 | 6 and 7 | 717 |
| 9 | Transplant recipients/ or Transplantation, Homologous/ae or Transplantation Immunology/ae, ci or ((transplant* or graft* or post-transplant* or allograft* or homograft*) and (Islands of Langerhans or Islands of Pancreas or Islets of Langerhans or Pancreatic Islet* or islet* or kidney? or renal* or nephr* or liver? or hepat* or post-liver* or organ* or tissue* or homologous* or immunolog* or immun* respon*)).mp. | 653332 |
| 10 | 8 and 9 | 81 |
| 11 | ("17436852" or "23862401").ui. -key article PMIDs | 2 |
| 12 | 10 and 11 – Validation only | 2 |
| 13 | limit 10 to english language | 71 |
| 14 | remove duplicates from 13 | 71 |

Table S2: Review of Cases of Atopic Dermatitis associated with Tacrolimus Therapy

| Reference | Age at AD onset  (years) | Sex | Type of Transplant | Age at Transplant (years) | Immuno-suppression Induction | Additional Immuno-  suppressive Agent | Time from Transplant to AD diagnosis (months) | AD diagnosis criteria used | AD management: non-pharmacologic | AD management: pharmacologic | Tacrolimus therapy decision | Reported comments on skin outcomes |
| --- | --- | --- | --- | --- | --- | --- | --- | --- | --- | --- | --- | --- |
| Cullison, 2018 | 0.5 | M | cardiac | 0.083 | NR | MMF(10-15 mg/kg/d) | 5 | NR | yes | TCS, topical CNI, topical antibiotic, antiviral, IVIG | dose decreased; sirolimus & prednisolone added | "immediate" improvement in skin once oral tacrolimus dose was reduced |
|  | 4 | M | cardiac | 0.25 | NR | Azathioprine (1.5 mg /kg/d) | 45 | NR | yes | topical and systemic antibiotics, TCS, topical CNI | Stopped tacrolimus +Prednisone,  Started sirolimus & MMF | cleared after systemic tacrolimus discontinuation |
|  | 0.5 | M | cardiac | 0.25 | NR | MMF (20-26 mg/kg/d) | 2 | NR | yes | TCS, topical CNI | no change | "marginally controlling his eczema" |
| Bumbacea, 2013 | 6 | M | parental living donor liver | NR | NR | NR | 24 | Hanifin and Rajka | yes | TCS, H1-antihistamine | NR | NR |
| Ludriksone, 2020 | 38 | F | orthotopic liver | 22 | NR | Cyclosporine, discontinued | 9 | Hanifin and Rajka + SCORAD of 81 | yes | TCS, topical Cis, emollients, repeated systemic steroids before adding Dupixent | no change | rapid improvement after starting Dupilumab (600 mg initially, then 300 mg SC q 2 weeks) |
| Ponte, 2007 | 43 | F | allogeneic from multiorgan donors | 43 | Infliximab | Sirolimus & 5 dose induction course of Dacllizumab | 12.3 | NR | yes | TCS, topical CNIs, antihistamines, phototherapy | changed to MMF | "within 2 weeks" from tacrolimus withdrawal, the patient reported significant improvement of rash/pruritus |
| Obayashi, 2015 | 1.5 | F | living-donor liver | 1 | NR | Prednisolone, discontinued | NR | NR | NR | NR | changed to CsA | "AD improved markedly" |
| Horino, 2023 | 1 | F | unrelated cord blood | NR | TBI 3Gy VP-16 Flu Mel ATG | short term methotrexate, temporary prednisolone | 9 | UK Working Party's for AD | yes | TCS, antihistamines, 0.5% delgocitinib ointment | no change reported | reduction in EASI, BSA, and NRS. |
|  | 15 | M | unrelated bone marrow |  | TBI 12Gy VP-16 Cy | short term methotrexate, temporary prednisolone | 14 | UK Working Party's for AD | yes | TCS, antihistamine | no change reported | reduction in EASI, BSA, and NRS scores. |
|  | 7 | M | related bone marrow |  | TAI 3Gy Cy Flu ATG | short term methotrexate, temporary prednisolone | 5 | UK Working Party's for AD | yes | TCS, antihistamine | no change reported | reduction in EASI, BSA, and NRS. |
|  | 16 | M | unrelated bone marrow |  | TBI 3Gy Flu Mel ATG | short term methotrexate | 2 | UK Working Party's Diagnostic Criteria for AD | yes | TCS, antihistamine | no change reported | reduction in EASI, BSA, and NRS. |
|  | 10 | F | related bone marrow |  | TBI 3Gy Flu Mel ATG | short term methotrexate | 6 | UK Working Party's Diagnostic Criteria for AD | yes | TCS, antihistamine | no change reported | reduction in EASI, BSA, and NRS. |
| Machura  /2015 | 3 | M | orthotopic heart | 0.5 | NR | MMF, discontinued; prednisone | NR | NR | yes | TCS, antihistamines, anti-scabies, anti-microbials | changed to CsA then back to tacrolimus | NR |

TCS: topical steroid, CsA cyclosporine, CNI: calcineurin inhibitor, NR: not reported, ATG: anti-Thymocyte antiglobulin

EASI: Eczema Assessment Severity Index, BSA: Body Surface area, NRS: numerical rating score (for itch)
